# A statistical normalization method and differential expression analysis for RNA-seq data between different species

**DOI:** 10.1186/s12859-019-2745-1

**Published:** 2019-03-29

**Authors:** Yan Zhou, Jiadi Zhu, Tiejun Tong, Junhui Wang, Bingqing Lin, Jun Zhang

**Affiliations:** 1 College of Mathematics and Statistics, Institute of Statistical Sciences, Shenzhen University, Shenzhen, 518060 China; 2 Department of Mathematics, Hong Kong Baptist University, Kowloon Tong, Hong Kong; 3 School of Data Science, City University of Hong Kong, Kowloon Tong, Hong Kong

**Keywords:** RNA-seq, Hypothesis test, Normalization, Differential expression, Orthologous genes

## Abstract

**Background:**

High-throughput techniques bring novel tools and also statistical challenges to genomic research. Identifying genes with differential expression between different species is an effective way to discover evolutionarily conserved transcriptional responses. To remove systematic variation between different species for a fair comparison, normalization serves as a crucial pre-processing step that adjusts for the varying sample sequencing depths and other confounding technical effects.

**Results:**

In this paper, we propose a scale based normalization (SCBN) method by taking into account the available knowledge of conserved orthologous genes and by using the hypothesis testing framework. Considering the different gene lengths and unmapped genes between different species, we formulate the problem from the perspective of hypothesis testing and search for the optimal scaling factor that minimizes the deviation between the empirical and nominal type I errors.

**Conclusions:**

Simulation studies show that the proposed method performs significantly better than the existing competitor in a wide range of settings. An RNA-seq dataset of different species is also analyzed and it coincides with the conclusion that the proposed method outperforms the existing method. For practical applications, we have also developed an R package named “SCBN”, which is freely available at http://www.bioconductor.org/packages/devel/bioc/html/SCBN.html.

**Electronic supplementary material:**

The online version of this article (10.1186/s12859-019-2745-1) contains supplementary material, which is available to authorized users.

## Background

High-throughput techniques provide a high revolutionary technology to replace hybridization-based microarrays for gene expression analysis [1–3]. The next-generation sequencing has evoked a wide range of applications, e.g., splicing variants [4, 5] and single nucleotide polymorphisms [6]. In particular, RNA-seq has become an attractive alternative to detect genes with differential expression (DE) between different species, which is used to explore the evolution of gene expression levels in mammalian organs [7] and the effect of gene expression levels in medicine. As an example, gene expression analyses performed in model species such as mouse is commonly used to study human diseases [8], including cancer [9, 10] and hypertension [11].

For different species, several studies have emerged in the recent literature to compare the gene expression levels in different organisms using microarrays or RNA-seq data. Liu et al. [12] reported a systematic comparison of RNA-seq for detecting differential gene expression between closely related species. Lu et al. [13] developed some probabilistic graphical models and applied them to analyze the gene expression between different species. Kristiansson et al. [14] proposed a statistical method for meta-analysis of gene expression profiles from different species with RNA-seq data. For different species, the RNA-seq experiments will result in not only different gene numbers and gene lengths, but also different read counts, i.e., sequencing depths. To make the expression levels of orthologous genes comparable between different species, normalization is a crucial step in the data processing procedure.

**Fig. 1:**
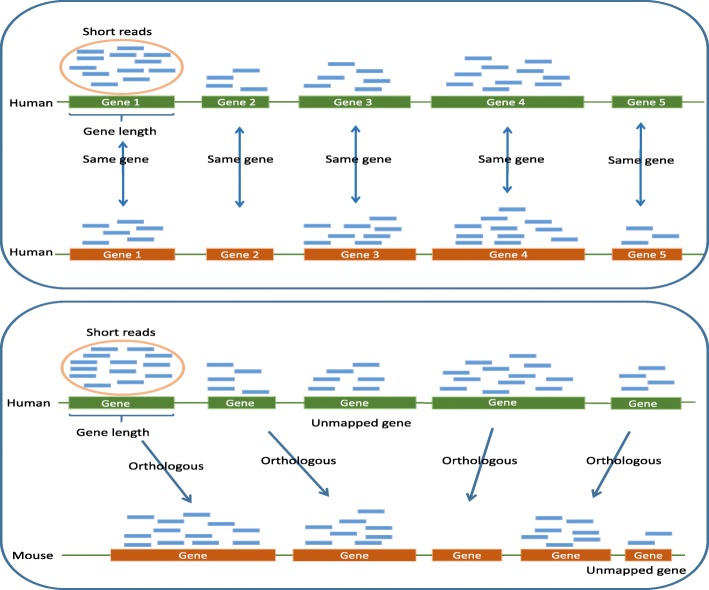
The first panel shows the same genes of different human samples, and the second panel shows the orthologous genes in human and mouse

The main purposes of normalization are to remove systematic variation and reduce noise in the data. In the case of one species (see the first panel of Fig. 1), various normalization methods have been developed in the last decade [15–18]. Mortazavi et al. [19] transformed RNA-seq data to reads per kilobase per million mapped (RPKM). Robinson et al. [20, 21] proposed a weighted trimmed mean of log-ratios method (TMM). Zhou et al. [22] developed a hypothesis testing based normalization (HTN) method by utilizing the available knowledge of housekeeping genes, and showed that the HTN method is more robust than TMM for analyzing RNA-seq data.

We note, however, that normalization of RNA-seq data with different species is more difficult than that with same species. For different species, we need to consider not only the total read counts but also the different gene numbers and gene lengths (see the second panel of Fig. 1). To the best of our knowledge, there are few studies in the literature for normalizing RNA-seq data with different species. As a routine method for normalization, one often standardizes the data with different species by scaling their total number of reads to a common value. For instance, Brawand et al. [7] used RPKM in Mortazavi et al. [19] to normalize RNA-seq data with different species. Specifically, they first identified the most conserved 1000 genes between species and then assessed their median expression levels in each species among the genes with expression values in the interquartile range for different species. Lastly, they derived the scaling factors that adjust those median values to a common value.

In this paper, we extend the HTN method from the setting of same species to different species. As described in Zhou et al. [22], HTN is a normalization method under different sequence depths for same species, and its performance outperforms other normalization methods. Based on the hypothesis testing framework, it transforms the problem to finding the scaling factor in normalization. By utilizing the available knowledge of housekeeping genes, it achieves the optimal scaling factor by minimizing the deviation between the empirical and nominal type I errors. However, HTN cannot be directly applied to RNA-seq data with different species, mainly because the assumption of the same numbers and lengths. For the setting of different species, we develop a scale based normalization (SCBN) method by utilizing the available knowledge of conserved orthologous genes and the hypothesis testing framework. Here, we use conserved orthologous genes for different species instead of housekeeping genes. It is noted that the normalization scaling factor is stable in both simulation studies and real data analysis.

The rest of the paper is organized as follows. We first propose the new SCBN method in “Materials and methods” section. We then conduct simulation studies to assess the performance of the SCBN method and also compare it with the existing method in “Simulation studies” section. In “Real data analysis” section, we apply the SCBN method to a real dataset with human and mouse to demonstrate its superiority over the existing method. The paper is concluded in “Discussion” section with some discussions and future work.

## Materials and methods

In the following section, we propose a novel normalization method for RNA-seq data with different species by utilizing the available knowledge of conserved orthologous genes and the hypothesis testing framework.

### Notations and model

Let *G*={*g*_1_,*g*_2_,…,*g*_*n*_} be the complete set of genes from two different species, and *G*_0_ be the set of one-to-one orthologous genes that are to be tested for differential expression. For species *t*=1 or 2, let $X_{g_{k}t}$ be the random variable that represents the count of reads mapped to the orthologous gene *g*_*k*_∈*G*_0_, and $X_{g_{k}t}$ be the observed value of $X_{g_{k} t}$. Accordingly, the total number of orthologous reads for species *t* is $N_{t}=\sum _{g_{k} \in G_{0}}x_{g_{k}t}$. For ease of presentation, our normalization method is presented for the setting of one sample in each species only. Our proposed method, however, can be readily extended to more general settings including multiple samples for each species. For gene *g*_*k*_ in species *t*, we consider the mean model:

1 $$ E(X_{g_{k}t}) = \frac{\mu_{g_{k}t}L_{g_{k}t}}{S_{t}}N_{t},  $$

where $\mu _{g_{k}t}$ is the true expression level, $L_{g_{k}t}$ is the true gene length, and $S_{t}=\sum _{g_{k} \in G_{0}}\mu _{g_{k}t}L_{g_{k}t}$ is the total expression output of all orthologous genes in species *t*. Note that, since $L_{g_{k}t}$ is often different between species, we have included it in model (1) to alleviate the bias in gene length.

### Novel normalization method

We propose a novel normalization method by employing the available knowledge of conserved orthologous genes and the hypothesis testing framework. Specifically, we choose a scale to minimize the deviation between the empirical and nominal type I errors in RNA-seq data based on the hypothesis test.

To detect differential expressions of orthologous genes between two species, for each *g*_*k*_∈*G*_0_, we consider the hypothesis

$$\begin{array}{@{}rcl@{}} H_{0}^{g_{k}}: \mu_{g_{k}1}=\mu_{g_{k}2}\quad \text{versus} \quad H_{1}^{g_{k}}: \mu_{g_{k}1}\neq \mu_{g_{k}2}. \end{array} $$

We further assume that the reads mapped to the orthologous genes are Poisson random variables with $\lambda _{g_{k}1}=E(X_{g_{k}1})$ and $\lambda _{g_{k}2}=E(X_{g_{k}2})$. Then under model (1), the hypothesis is equivalent to

2 $$ {{} \begin{aligned} H_{0}^{g_{k}}\!:\!\lambda_{g_{k}1}\,=\,\frac{L_{g_{k}1}}{L_{g_{k}2}}\frac{N_{1}}{N_{2}}c\lambda_{g_{k}2}~ \text{versus}\ \ H_{1}^{g_{k}}\!:\!\lambda_{g_{k}1}\!\neq\!\frac{L_{g_{k}1}}{L_{g_{k}2}}\frac{N_{1}}{N_{2}}c\lambda_{g_{k}2},  \end{aligned}}  $$

where *c*=*S*_2_/*S*_1_ is the scaling factor for normalization.

Given that $X_{g_{k}1}+X_{g_{k}2}=n_{g_{k}}$ with $n_{g_{k}}$ a fixed integer, the random variable $X_{g_{k}1}$ follows a binomial distribution with the conditional probability density function as

$${\begin{aligned} &P\left(X_{g_{k}1}= x_{g_{k} 1}\big|{X_{g_{k}1}+X_{g_{k}2}}=n_{g_{k}}\right) \\&\quad=\frac{n_{g_{k}}!}{x_{g_{k}1}!\left(n_{g_{k}}-x_{g_{k}1}\right)!} \left(p_{0}^{g_{k}}\right)^{x_{g_{k}1}} \left(1-p_{0}^{g_{k}}\right)^{n_{g_{k}}-x_{g_{k}1}}, \end{aligned}} $$

where

$$p_{0}^{g_{k}}=\frac{\lambda_{g_{k}1}}{\lambda_{g_{k}1}+\lambda_{g_{k}2}}= \frac{ {g_{k}1}N_{1}}{L_{g_{k}2}N_{2}+{cL}_{g_{k}1}N_{1}}$$

is the probability of success under the null hypothesis of (2). For the above model, the *p*-value of the test is

3 $$\begin{array}{*{20}l} p_{g_{k}}(c)&=P\left(| X_{g_{k}1}-n_{g_{k}} p_{0}^{g_{k}}|\geq | x_{g_{k}1}-n_{g_{k}} p_{0}^{g_{k}}|\big|n_{g_{k}}\right)\notag \\ &=P\left(|(1+\frac{L_{g_{k}1}}{L_{g_{k}2}}\frac{N_{1}}{N_{2}}c\right)X_{g_{k}1}-\frac{L_{g_{k}1}}{L_{g_{k}2}}\frac{N_{1}}{N_{2}}{cn}_{g_{k}}| \geq \\ &~~~~~\left|\left(1+\frac{L_{g_{k}1}}{L_{g_{k}2}}\frac{N_{1}}{N_{2}}c)x_{g_{k}1}-\frac{L_{g_{k}1}}{L_{g_{k}2}}\frac{N_{1}}{N_{2}}{cn}_{g_{k}}|\right|n_{g_{k}}\right).  \end{array} $$

Note that the *p*-value in (3) is a function of the scaling factor *c* under the condition $X_{g_{k}1}+X_{g_{k}2}=n_{g_{k}}$. To search for the optimal *c* for normalization, we apply the following two questions as criteria. (i) Does the normalization method improve the accuracy of DE detection, i.e., whether or not it will decrease the false discovery rate (FDR) of the tests? (ii) Does the normalization method result in a lower technical variability or specificity? For multiple testing, Storey [23] pointed out that different hypothesis tests will result in different significant regions. To transform these tests into a common space, the *p*-value is a natural way to do so with respect to the positive false discovery rate (pFDR). By taking the number of set *G*_0_ identical hypothesis tests, the pFDR is defined as follows:

4 $$\begin{array}{*{20}l} {} \text{pFDR}_{g_{k}}&=\!\frac{P(H_{0};c)P(R_{g_{k}}\mid H_{0}; c)}{P(R_{g_{k}};c)} \\ &\,=\,\frac{P(H_{0};c)P(R_{g_{k}}\mid H_{0};c)}{\!P(H_{0};c)P(R_{g_{k}}\mid H_{0};c)\,+\,P(H_{1};c)P(R_{g_{k}}\mid H_{1};c)\!},  \end{array} $$

where *α* is the significance level and $R_{g_{k}}=\{p_{g_{k}}(c) < \alpha \}$ is the rejection region. By (4), the pFDR of gene *g*_*k*_ is a function of both *α* and *c*. Given the values of *α* and *c*, we can apply the empirical distributions to estimate $P(R_{g_{k}}|H_{0};c)$ and $P(R_{g_{k}}|H_{1};c)$. Let *V*_0_ and *V*_1_ be the sets of non-DE genes and DE genes in *G*_0_, respectively. Then, $\text {pFDR}_{g_{k}}(\alpha ;c)$ can be estimated as

$$\begin{array}{@{}rcl@{}} \widehat{\text{pFDR}}_{g_{k}}=\!\frac{P(H_{0};c){\widehat P}(R_{g_{k}}\mid H_{0};c)}{P(H_{0};c){\widehat P}(R_{g_{k}}\mid H_{0};c)+P(H_{1};c){\widehat P}(R_{g_{k}}\mid H_{1};c)\!}, \end{array} $$

where

$${\widehat P}(R_{g_{k}} \mid H_{0};c)=\frac{1}{n_{0}}\sum_{g_{k}\in V_{0}}I(p_{g_{k}}(c)<\alpha|H_{0};c)$$

for any *g*_*k*_∈*V*_0_, and

$${\widehat P}(R_{g_{k}}\mid H_{1};c)=\frac{1}{n_{1}}\sum_{g_{k}\in V_{1}}I(p_{g_{k}}(c) <\alpha|H_{1};c)$$

for any *g*_*k*_∈*V*_1_, where *I*(·) is the indicator function, and *n*_0_ and *n*_1_ represent the cardinalities of *V*_0_ and *V*_1_, respectively.

When all non-DE genes in *V*_0_ are given, we can perform our new normalization by determining the optimal scaling factor that minimizes the value of pFDR. For real data, however, it is not uncommon that only a small proportion of non-DE genes are known a priori by background knowledge. In this paper, we assume that a set of conserved orthologous genes between species are given in advance, which may either be reported in other studies or be selected by a certain biological measure [7, 24]. For the given set *H* of conserved orthologous genes that are considered as non-DE genes for its stability between species, we search for the optimal scaling factor by minimizing the deviation between the empirical and nominal type I errors. Let *m* be the number of genes in the set *H*. Given the true value of *c*, the *p*-values of the tests for the conserved orthologous genes follow a uniform distribution on interval (0,1). That is, for the specified *α* and *c*, the value of $\sum _{g_{k} \in H}(1/m)I(p_{g_{k}}(c)<\alpha |H_{0};c)$ should be around the nominal level at *α*. In our method, we define the optimal scaling factor as *c*_opt_ that minimizes the objective function $\mid \sum _{g_{k} \in H}(1/m)I(p_{g_{k}}(c)<\alpha |H_{0};c)-\alpha \mid $; that is,

5 $$\begin{array}{*{20}l} c_{\text{opt}}=\underset{c>0}{\text{argmin}} \big|\sum_{g_{k} \in H}\frac{1}{m}I(p_{g_{k}}(c)<\alpha|H_{0};c)-\alpha \big|.  \end{array} $$

Finally, to estimate the optimal scaling factor defined in (5), we apply a grid search method and denote the best estimate as $\hat c_{\text {opt}}$. For convenience, we refer to the proposed scale based normalization method as the SCBN method.

## Simulation studies

For a fair comparison, we generate the simulation datasets following the settings in Robinson et al. [20], but with the structure of different species rather than same species. For different species, we consider different sequencing depths and lengths of orthologous genes to generate the datasets, including DE genes, non-DE genes and unmapped genes for two species to mimic the real scenario. The unmapped genes represent those genes that exist only in one species. They are different from the unique genes, representing those orthologous genes that exist in both species but are expressed in only one of them. After setting the number of unique genes and unmapped genes, proportion, magnitude and direction of DE genes between two species, we randomly generate the rate of a gene expression level to the output of all the orthologous genes from a given empirical distribution of real counts. We set the expected values of the Poisson distributions from model (1), and then randomly generate simulation datasets from the respective distributions.

We first evaluate the stability of the proposed SCBN method for the fixed parameters. In Study 1, we compare the false discovery number of the SCBN method and the median method with different number of conserved genes. We set 10% of the orthologous genes as DE genes at the 1.2-fold level; of those DE genes, 90% are up-regulated in the second species, and we set the number of unique genes as 1000 and 2000 for two species, respectively. Besides, we set 2000 and 4000 unmapped genes for two species. With the fixed parameters, we consider the cases where the number of conserved orthologous genes varies from 50 to 1000. In Study 2, the parameters are the same as those in Study 1 except that the fold level of DE genes is increased to 1.5, and we select 1000 conserved genes in each experiment. Then, we investigate the stability of the proposed method when the rates of noise in conserved genes increase from 0 to 0.6 with step size 0.1. In Study 3, we consider the adjusted M versus A plots in Lin et al. [20] to compare the scaling factors of two normalization methods when the rate of noise in conserved genes equal to 0 and 0.4. In this paper, the rate of noise means the proportion of DE genes in all of the conserved genes. To make it more obvious, we adjust the parameters with 20% DE genes at the 8-fold level, and 70% are up-regulated in the second species. The unique genes and unmapped genes are the same as before. In Study 4, we test the stability of the SCBN method by choosing different *p*-values as cutoff. In this study, we consider the cutoff values varying from 0.0001 to 0.6. The parameters are the same as those in Study 1 except that 40% of genes are differentially expressed.

Next, we investigate the performance of the SCBN method with several criteria, including the false discovery number, precision, sensitivity and *F*-score, which were also adopted in [25]. In Studies 5 and 6, the parameters are kept the same as those in Study 2. In Study 5, the false discovery number of the two normalization methods are shown with different rates of noise in conserved genes, ranging from 0 to 0.5. In Study 6, we compare the precision, sensitivity and *F*-score for the two methods. The precision denotes the rate of true positives in all the predicted positives, the sensitivity represents the rate of true positives in all real positives, and the *F*-score is a metric to overview both the precision and sensitivity. Here, we take 0.01 as the *p*-value cutoff.

In Study 7, we compare the performance of the two methods for different rates of DE genes in all orthologous genes. We set the fold change of DE genes as 1.5, the rate of noise in conserved genes as 0.2, and the rates of DE genes varying from 0.1 to 0.6. Other parameters are kept the same as those in Study 4.

**Fig. 2:**
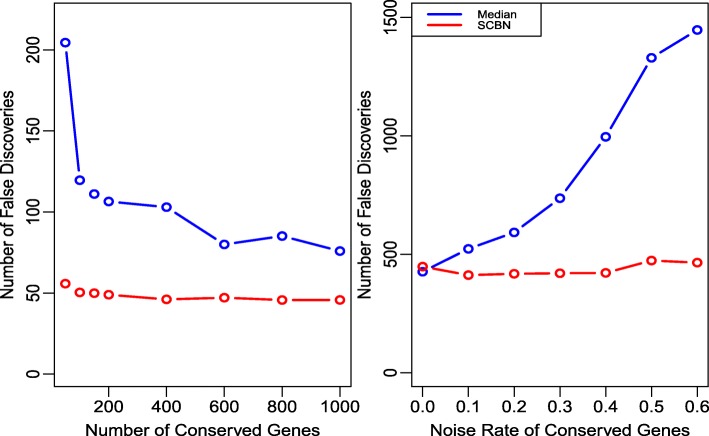
The left panel is the false discovery number of the median and SCBN methods with different number of conserved genes. The right panel is the false discovery number of the two methods with different rates of noise in conserved genes

**Fig. 3:**
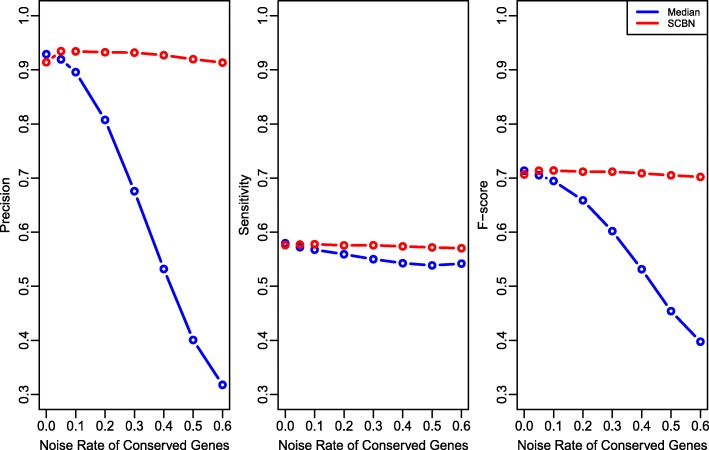
Precision (left), sensitivity (middle) and *F*-score (right) values of two normalization methods with various rates of noise

For each simulated dataset, we compare the false discovery number, which are computed by repeating the simulation 100 times, while there are time consuming in each repeat, and averaging over all the repetitions. We report the stability of the SCBN method with various parameters in Fig. 2. Figure 3 compares the SCBN method to the median method with precision, sensitivity and *F*-score criteria. The Additional file 1 compares the false discovery number with different rates of noise in the selected conserved genes.

The left panel of Fig. 2 (Study 1) shows that the false discovery number is reduced as the number of conserved genes increases. Whereas the false discovery number of the median method increase drastically when conserved genes become less, the SCBN method is much more robust to the number of conserved genes. Furthermore, the SCBN method performs much better than the median method for each number of conserved genes. As shown in the right panel of Fig. 2 (Study 2), the false discovery number of the SCBN method keeps stable, but that of the median method increases gradually as the rate of noise increases. From these two studies, we can see that the SCBN method is more robust than the median method, especially when the number of conserved gene is small, or the rate of noise is large.

In Study 3, the two scaling factors are presented in Additional file 2. From the left panel, the lines of the two normalization methods are close when conserved genes do not include noise. However, as the rate of noise equals to 0.4, the right panel shows the scaling factor of the SCBN method is much closer to the center of non-DE genes. Additional file 3 presents the result of Study 4, which demonstrates the choice of *p*-value cutoffs has no impact on the results of the SCBN method.

In Study 5, we investigate the overall situations of false discoveries changed with different rates of noise. The results are shown in Additional file 3, which shows that the two normalization methods have a similar performance when all selected conserved genes are non-DE genes. However, the SCBN method outperforms the median method when the rate of noise becomes larger than 0.1. Hence, we conclude that the SCBN method performs significantly better than the median method when moderate-to-large rates of noise are presented.

Figure 3 shows the experimental results of precision, sensitivity and *F*-scores. Since *F*-score is the harmonic mean of precision and sensitivity, it is clear that the SCBN method has overall better performance as it achieves higher *F*-scores in most cases. As we can see from the plots, when the rate of noise is less than 0.1, the values of sensitivity and *F*-score for two normalization methods are very close. The median method performs slightly better than the SCBN method in precision when conserved genes have no noise or small noise, but its precision decreases enormously with noise increased. For instance, the precisions of the median method are 0.93, 0.68 and 0.32 with conserved genes have 0, 30% and 60% of DE genes. The SCBN method has precision values 0.91, 0.93 and 0.91, respectively. It is evident that the median method depends greatly on the selected conserved genes, including the number and purity of conserved genes. On contrary, conserved genes have much less impact on the performance of the SCBN method.

**Fig. 4:**
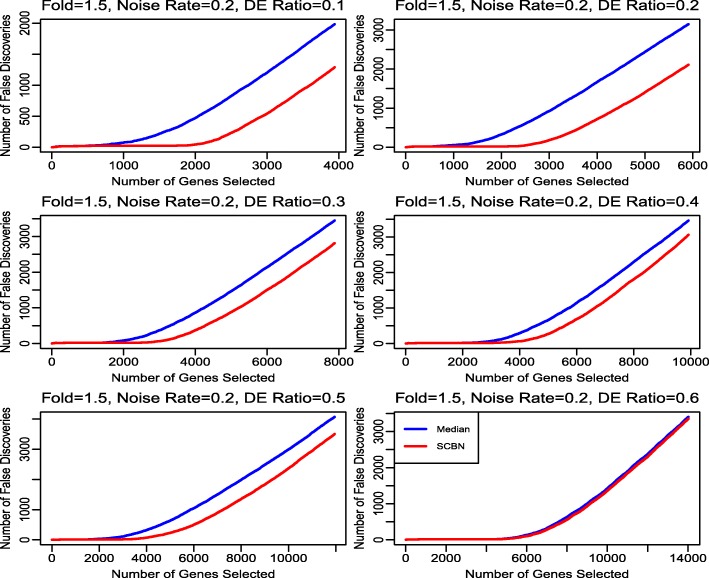
The false discovery number of two normalization methods with DE genes at the rates of 0.1, 0.2, 0.3, 0.4, 0.5 and 0.6, respectively

In Study 7, we focus on the impact of the rate of DE genes on two normalization methods. Figure 4 shows that the SCBN method outperforms the median method for various rates of DE genes, especially when the rate of DE genes is not too large. The result implies that the SCBN method is more sensitive to identify less fold of DE genes than that of the median method.

## Real data analysis

We illustrate the usefulness of the SCBN method in real dataset by the study of Brawand et al. [7]. The real data were obtained by using the mRNA-seq Sample Prep Kit (Illumina) platform with paired-end sequencing, and using TopHat and Bowtie softwares to map the reads. The dataset consists of two groups of orthologous transcripts in human and mouse, with respective transcripts lengths and counts of reads (see Additional file 4 for details). We refer to the human transcripts (GRCh38.p10) and the mouse transcripts (GRCm38.p5) in Ensembl database, which is available at http://asia.ensembl.org/biomart/martview/4e1666ae95e54c2f42ae0402dad82e73.

**Fig. 5:**
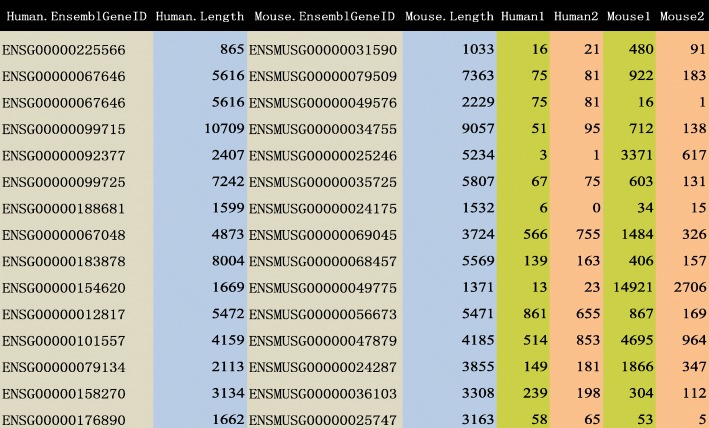
The RNA-seq data of orthologous transcripts in human and mouse

There are a total of 63967 transcripts in human and 53946 transcripts in mouse, 27779 of which are orthologous transcripts (see the right panel of Fig. 1). By excluding the unmatched, duplicated and unexpressed transcripts, there are 19330 available orthologous transcripts. Figure 5 shows the expressions of several orthologous transcripts in human and mouse.

As shown in Fig. 1, unlike the case of same species where the number and lengths of genes are equal to each other, different species have different gene number and thus different gene lengths. Regarding the different lengths of orthologous transcripts, only 105 transcripts or only 0.54% of all transcripts, have the same lengths between human and mouse in Additional file 5. The average difference of the transcripts lengths between two species is 1039, and the maximum is 21666 in Additional file 6. The evolutionary process of the eukaryotic genome includes events such as duplication and recombination, which creates complicated relationships among genes. As a consequence, the normalization methods for same species may not provide a satisfactory performance or may not even be applicable for different species. The challenges of normalization between different species are mainly due to the different lengths of orthologous genes and the different sequencing depths due to the different platforms.

We get the conserved orthologous genes with a three-step procedure. First, we confirm the orthologous transcripts between human and mouse, by using the BioMart function in the Ensembl to search all human transcripts and filtering out the genes that do not exist in mouse. Second, according to the orthology quality-controls criterion, we sort the data from the most conserved to the least. Third, we select the 143 most conserved orthologous transcripts between human and mouse and list them in Additional file 7.

The most conserved 500 or 1000 orthologous transcripts are likely non-DE transcripts between two species, and we compare the two methods with the first group data. First, we select the most 500 or 1000 conserved transcripts with the above steps, and then use the two methods to normalize the sequence data with the 143 conserved transcripts. Next, we calculate *p*-values (see Additional file 8) with adjusted sage.test function. Last, we get DE transcripts between human and mouse with *p*-value cutoff 10^−6^, which are shown in Table 1. Among the most conserved 500 or 1000 orthologous transcripts, 332 and 647 of them are detected as DE transcripts by using the SCBN method, in which 48% and 46% significantly higher in human, whereas the median method detects 351 and 697 DE transcripts, in which 32% and 29% significantly higher in human. For all orthologous transcripts, the SCBN method detects 9662 DE transcripts, and the median method detects 9910 DE transcripts. Assuming that the most conserved 500 orthologous transcripts are non-DE transcripts, there are 351 false detected DE transcripts with the median method and 332 false detected DE transcripts with the SCBN method. Then the FDR of the median method is 0.035, which is larger than 0.034 of the SCBN method. For the 1000 conserved transcripts, we get a similar result that the FDR of the median method (0.070) is also larger than that of the SCBN method (0.067). Therefore, the FDRs of the SCBN method are generally smaller than those of the median method.

**Table 1:** The number of DE genes between human and mouse at a cutoff *p*-value <10^−6^ for the median and the SCBN methods

	Median	SCBN	Overlap
Higher in human	4370	5824	2610
Higher in mouse	5540	3838	2184
Total	9910	9662	4794
Top conserved genes (500)			
Higher in human	112	159	56
Higher in mouse	239	173	119
Total	351	332	175
Top conserved genes (1000)			
Higher in human	201	300	87
Higher in mouse	496	347	240
Total	697	647	327

**Fig. 6:**
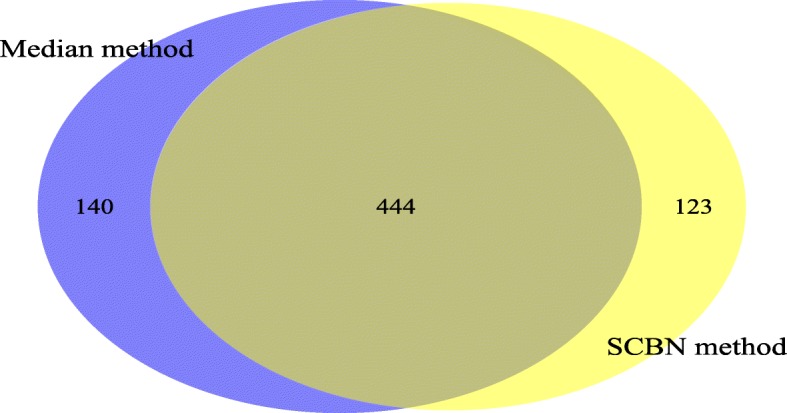
The common genes and the unique DE genes detected by two normalization methods

Next, we compare the accuracy of the two normalization methods by looking deeper into the biological function. We apply the SCBN method to detect the most significant 1000 DE transcripts for each pair comparison between human and mouse, that is the smallest 1000 *p*-values for each comparison, among which 567 are common. Also, the median method detects 584 common DE transcripts for two species. Figure 6 shows the common DE transcripts and the unique DE transcripts of the two normalization methods. For the unique transcripts, we refer to NCBI [26] to find out which genes are associated with evolution or illness. There are 48 of 123 (39.02%) DE transcripts, which are related to evolution or illness with the SCBN method, and 43 of 140 (30.71%) DE transcripts are related to evolution or illness with the median method. Specifically, among the unique DE transcripts detected by the SCBN method, we find that ‘ENSG00000102316’ is involved in breast cancer and melanoma, ‘ENSG00000152137’ is involved in the regulation of cell proliferation, apoptosis, and carcinogenesis, and ‘ENSG00000135744’ is associated with the susceptibility to essential hypertension, and can cause renal tubular dysgenesis, a severe disorder of renal tubular development. Mutations in gene ‘ENSG00000152137’ have been associated with different neuromuscular diseases, including the Charcot-Marie-Tooth disease. We note, however, that above genes are not included in the 584 most significant DE transcripts detected by the median method. More details are presented in Additional file 9. The results show that the SCBN method provides a more accurate normalization than the median method in real data analysis.

## Discussion

Detecting DE genes between different species is an effective way to identify evolutionarily conserved transcriptional responses. For different species, the RNA-seq experiments will result in not only different read counts, but also different numbers and lengths of genes. To make the expression levels of orthologous genes comparable between different species, normalization is a crucial step in the process of detecting DE genes. This is in sharp contrast to the case of same species, where the numbers and lengths of genes are equal to each other. The existing normalization methods for same species may not provide a satisfactory performance or may not even be applicable for RNA-seq data with different species. Therefore, developing new normalization methods for RNA-seq data with different species is extremely urgent.

In this paper, we propose a scale based normalization (SCBN) method between different species for RNA-seq data. For the SCBN method, it could be used to deal with non-negative and discrete RNA-seq counts. Therefore, the proposed method is suitable to deal with paired-end and single-read sequencing data by using the most widely used sequencing technologies, including Illumina (Solexa) sequencing, Roche 454 sequencing, Ion torrent: Proton/ PGM sequencing and SOLiD sequencing. The SCBN method is also compatible with two main types of RNA-seq mappers, including unspliced aligners and spliced aligners. Two main contributions of our work are: (i) dealing with RNA-seq data with two different species, which have different lengths of genes and sequencing depths, and (ii) employing the hypothesis testing approaches to search for the optimal scaling factor, which minimizes the deviation between the empirical and nominal type I errors. From the simulation results, we find that the proposed SCBN method outperforms the existing median method, especially when the number of the selected conserved genes is small or the selected conserved genes involve a lot of noise. In real data analysis, we analyze an RNA-seq data of two species, human and mouse, and the results indicate that the SCBN method delivers a more satisfactory performance than the median method.

Compared to the RNA-seq data with same species, the normalization procedure between different species is much more complicated. Although the proposed method has largely improved the effectiveness to detect DE genes in some cases, we note that it may still not be able to provide a satisfactory performance when the rate of DE genes is very high in the whole samples. In addition, the unmatched genes and the relation of orthologous genes are not considered in the process of normalization between different species. This may call for a future work that develops new methods to further improve our current method.

## Additional files

Additional file 1 The false discovery number at the rates of noise in selected conserved genes being 0, 0.1, 0.2, 0.3, 0.4 and 0.5, respectively. (PDF 180 KB)

Additional file 2 M versus A plots of two normalization methods. (PDF 2457 KB)

Additional file 3 The scaling factors with different p-value cutoffs. (PDF 5 KB)

Additional file 4 Two groups of orthologous transcripts in human and mouse. (TXT 2450 KB)

Additional file 5 The length difference of the orthologous transcripts between human and mouse. (PDF 34 KB)

Additional file 6 The histogram of the length difference of the orthologous transcripts between human and mouse. (PDF 5 KB)

Additional file 7 143 most conserved orthologous transcripts between human and mouse and orthology quality-controls criterion. (CSV 9 KB)

Additional file 8 *p* −values and *q* −values for each orthologous transcripts. (XLSX 2587 KB)

Additional file 9 The details for 140 and 123 differentially expressed orthologous transcripts detected by the Median and the SCBN method respectively. (XLSX 24 KB)
