# Supplementary material for: A statistical normalization method and differential expression analysis for RNA-seq data between different species
Source: BMC Bioinformatics. 2019 Mar 29;20:163. doi: 10.1186/s12859-019-2745-1 (PMC6441199; doi:10.1186/s12859-019-2745-1)
Supplement: Supplementary file 1 — The false discovery number at the rates of noise in selected conserved genes being 0, 0.1, 0.2, 0.3, 0.4 and 0.5, respectively. (PDF 180 KB) [file 12859_2019_2745_MOESM1_ESM.pdf]

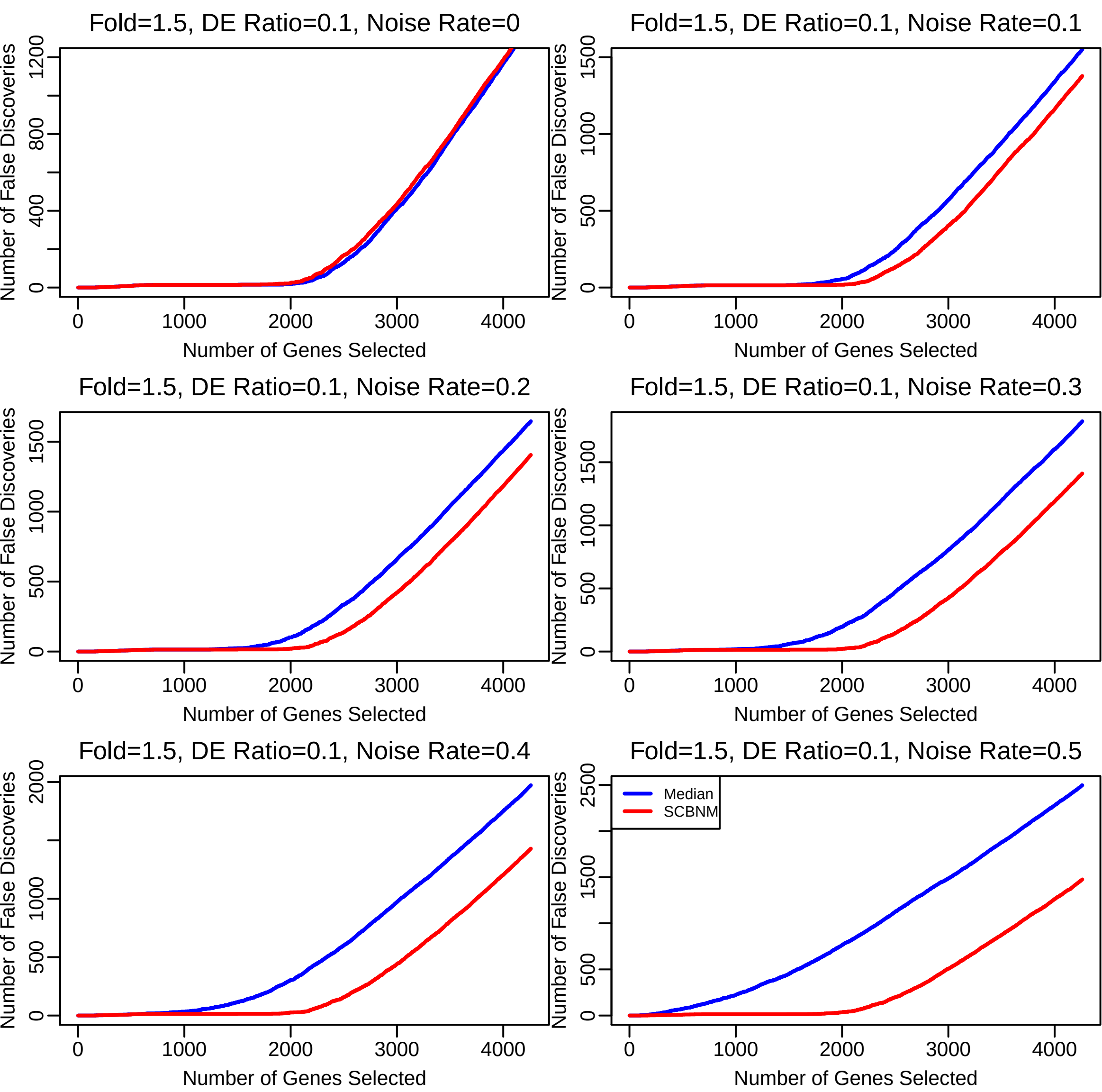
Fold=1.5, DE Ratio=0.1, Noise Rate=0
Fold=1.5, DE Ratio=0.1, Noise Rate=0.1
Fold=1.5, DE Ratio=0.1, Noise Rate=0.2
Fold=1.5, DE Ratio=0.1, Noise Rate=0.3
Fold=1.5, DE Ratio=0.1, Noise Rate=0.4
Fold=1.5, DE Ratio=0.1, Noise Rate=0.5
Number of False Discoveries
Number of Genes Selected
0
1000
2000
3000
4000
400
800
1200
500
1500
2500
Median
SCBNM
