# Supplementary figures and images for: A statistical normalization method and differential expression analysis for RNA-seq data between different species
Source: BMC Bioinformatics. 2019 Mar 29;20:163. doi: 10.1186/s12859-019-2745-1 (PMC6441199; doi:10.1186/s12859-019-2745-1)

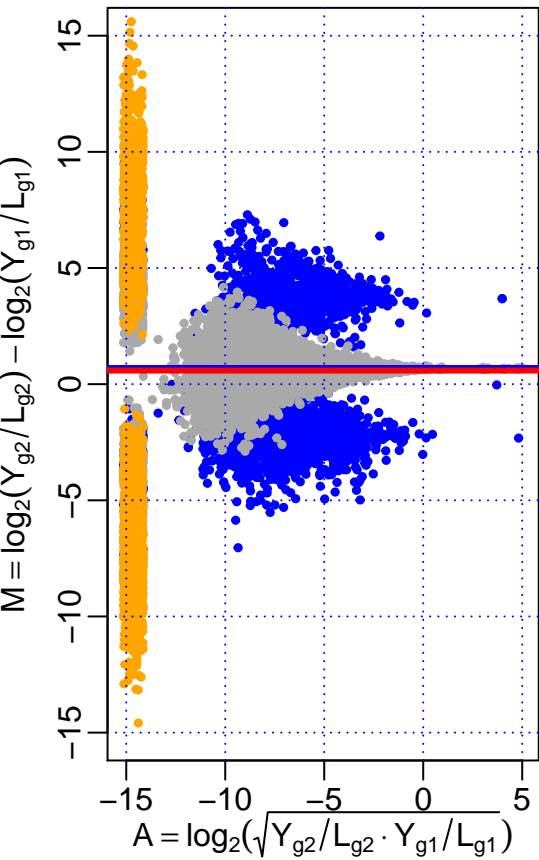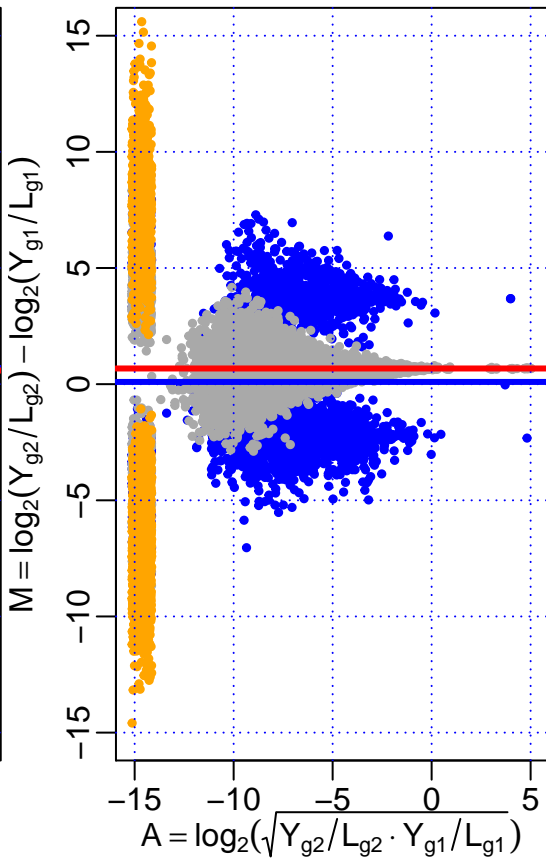

Supplement: Supplementary file 2 — M versus A plots of two normalization methods. (PDF 2457 KB) [file 12859_2019_2745_MOESM2_ESM.pdf]

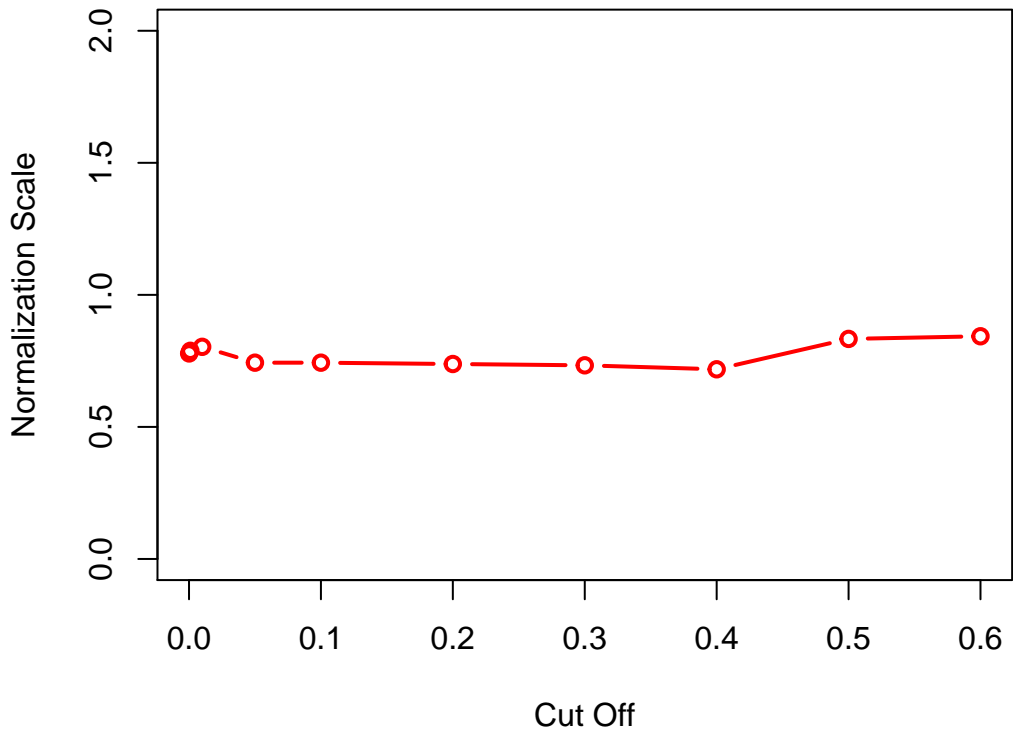

Supplement: Supplementary file 3 — The scaling factors with different p-value cutoffs. (PDF 5 KB) [file 12859_2019_2745_MOESM3_ESM.pdf]

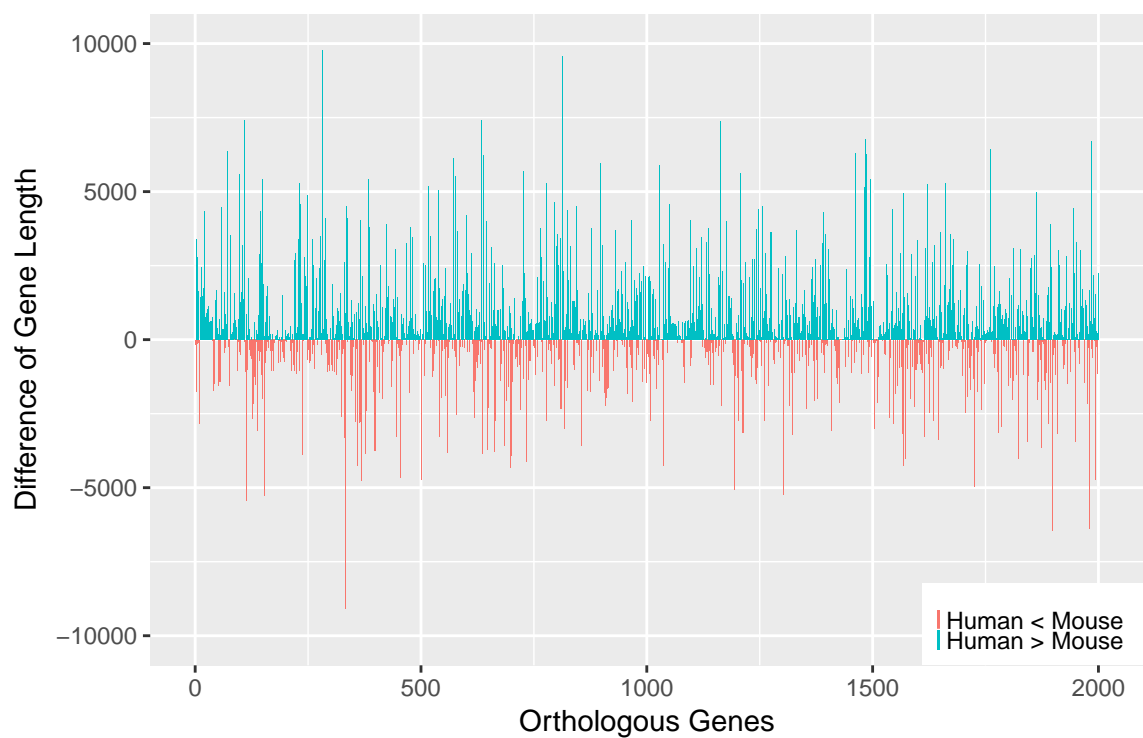

Supplement: Supplementary file 5 — The length difference of the orthologous transcripts between human and mouse. (PDF 34 KB) [file 12859_2019_2745_MOESM5_ESM.pdf]

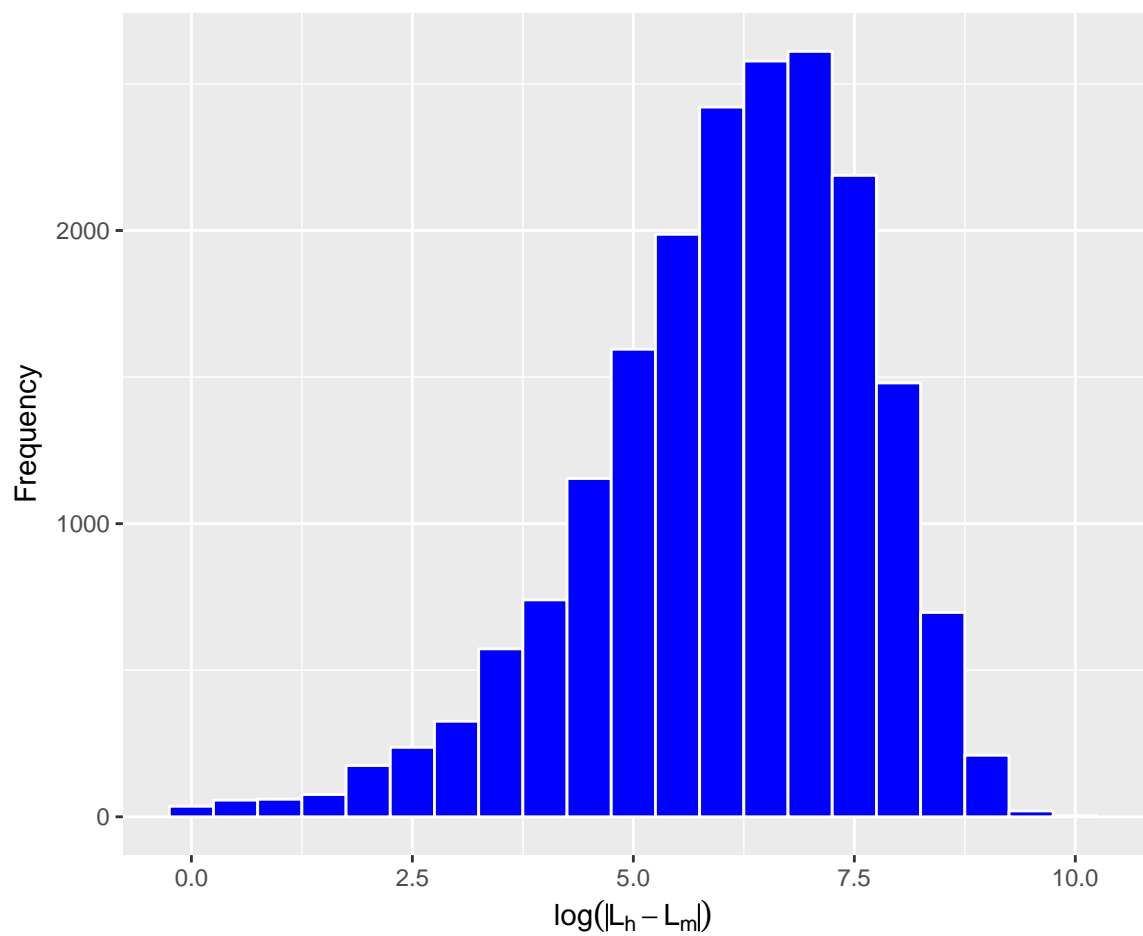

Supplement: Supplementary file 6 — The histogram of the length difference of the orthologous transcripts between human and mouse. (PDF 5 KB) [file 12859_2019_2745_MOESM6_ESM.pdf]
